# Recent Advances in Mechanical Reinforcement of Zwitterionic Hydrogels

**DOI:** 10.3390/gels8090580

**Published:** 2022-09-13

**Authors:** Weifeng Lin, Xinyue Wei, Sihang Liu, Juan Zhang, Tian Yang, Shengfu Chen

**Affiliations:** 1 Key Laboratory of Bio-Inspired Smart Interfacial Science and Technology of Ministry of Education, School of Chemistry, Beihang University, Beijing 100191, China; 2 Key Laboratory of Biomass Chemical Engineering of Ministry of Education, College of Chemical and Biological Engineering, Zhejiang University, Hangzhou 310027, China; 3 State Key Laboratory of Advanced Optical Communication Systems and Networks, Key Laboratory for Thin Film and Microfabrication of the Ministry of Education, UM-SJTU Joint Institute, Shanghai Jiao Tong University, Shanghai 200240, China; 4 Zhejiang Poly Pharm Co., Ltd., Hangzhou 311199, China

**Keywords:** zwitterionic hydrogels, mechanical properties, tough hydrogel, double network hydrogel, dual-cross-linked hydrogels, nanocomposite hydrogels, anti-fouling, mechanical energy dissipation

## Abstract

As a nonspecific protein adsorption material, a strong hydration layer provides zwitterionic hydrogels with excellent application potential while weakening the interaction between zwitterionic units, leading to poor mechanical properties. The unique anti-polyelectrolyte effect in ionic solution further restricts the application value due to the worsening mechanical strength. To overcome the limitations of zwitterionic hydrogels that can only be used in scenarios that do not require mechanical properties, several methods for strengthening mechanical properties based on enhancing intermolecular interaction forces and polymer network structure design have been extensively studied. Here, we review the works on preparing tough zwitterionic hydrogel. Based on the spatial and molecular structure design, tough zwitterionic hydrogels have been considered as an important candidate for advanced biomedical and soft ionotronic devices.

## 1. Introduction

Hydrogels, as composites of liquid water and solid polymer networks, have many potential applications in biomedicine engineering [1], biosensors [2], and flexible electrolytes [3]. However, their mechanical properties are limited [4]. The mechanical properties of hydrogels are directly related to the polymer network structure and its water content [5]. When water molecules are sufficiently bound to each hydrophilic polymer unit, the polymer network is fully swollen, and the interaction between the polymer chains is blocked by the hydration layer. Once the mechanical energy transferred to the polymer network exceeds the intrinsic fracture toughness, the network of the hydrogel breaks, and the structure collapses rapidly, revealing brittle. When water molecules can only combine with a small number of hydrophilic units to form a hydrated layer, the interaction between polymer chains takes on the function of mechanical energy dissipation, revealing toughness [6].

In the past two decades, the tough hydrogels, which were designed by structural design [7,8], molecular design [9], and multi-component combination [10], have been widely used in tissue engineering [11,12], flexible electrolytes [13,14], and viscous materials with Young’s modulus from several Pa to hundreds MPa [15,16]. Among them, zwitterionic hydrogels have attracted considerable attention due to their long-term anti-fouling properties in physiological environments and their ability to accelerate lithium salt dissociation, especially in biomedical engineering and solid electrolyte-based fields [17,18,19,20]. However, the very low Young’s modulus (a few KPa, similar to the brain or spinal cord) [13] and the rapid swelling in ionic solutions that ultimately leads to the destruction of polymer networks seem to indicate that the excellent application value of zwitterionic hydrogels is severely constrained by their poor mechanical properties.

Based on the current strengthening strategies of hydrogels, the design and application of toughness zwitterionic hydrogels have been widely studied. In this article, we classify the current mechanical strengthening strategies of hydrogels and review their contribution to zwitterionic hydrogels. We hope to show a more comprehensive illustration of the interaction between the molecules, structural design, and other work of zwitterionic hydrogels to further expand their application scenarios. It is worth noting that only zwitterionic polymers with both cationic and anionic functional groups in each side chain unit are discussed in this review [21,22]. Other zwitterionic polymers in a broad sense, such as polyamphiphilic electrolyte hydrogel with copolymerized equimolar cationic and anionic components are not discussed due to the potential loss of basic zwitterionic properties in multivalent ion solution [23,24].

## 2. Characteristics of Zwitterionic Hydrogels

Compared with the polymers hydrated by hydrogen bonds, the ionic hydration layer of the zwitterionic polymer has higher stability and degree of freedom [25]. On the one hand, the existence of a hydration layer significantly reduces the non-specific binding between proteins and the zwitterionic surface, endowing zwitterionic hydrogels with a unique ability to resist biological contamination [26] and avoid immune recognition [27]. On the other hand, it provides ion channels that can be used at lower temperatures, making zwitterionic hydrogels important candidates for antifreeze solid electrolytes [28,29]. In addition, zwitterionic materials can also modulate reactive oxygen species (ROS) and regulate inflammatory pathways by modulating macrophage polarization from M1 to M2 phenotype [30]. 

However, the strong hydration layer also hinders the formation of dipole–dipole interaction between zwitterionic units. Even the polymers with a similar charge density of cationic group and anionic group (pSBMA) [31], which is easier to form self-association and respond to external stimuli, the dipole–dipole interaction between zwitterionic polymer units cannot provide enough mechanical energy dissipation, resulting in the lack of mechanical properties of hydrogels. In addition, as the ion concentration increases, the counterions in the solution bind with the polyions, further destroying the dipole–dipole interaction between the zwitterionic units and extending the polymer chain [3,17]. The resulting zwitterionic hydrogels continue to swell, becoming brittle. This behavior of zwitterionic hydrogels is known as the anti-polyelectrolyte effect. From the application point of view, this poses serious problems: (1) as biomedical engineering materials, the mechanical properties of zwitterionic hydrogels can only match part of the soft tissues but are far from the requirements of stiff load-bearing tissues (fracture strength 1–200 MPa, elastic modulus 0.01–3 GPa) [32]; (2) as a quasi-solid electrolyte, zwitterionic hydrogels have to face a trade-off between swelling in ionic solutions and loss of mechanical properties [33].

Therefore, it is necessary to improve the mechanical properties of zwitterionic hydrogels using the available strengthening strategies. Due to the various nomenclature of zwitterionic polymers, in this review, we re-abbreviate the commonly used zwitterionic betaine according to the structure characteristics of zwitterionic units for distinguishing. SB stands for sulfobetaine, CB for carboxybetaine, MA for methacrylate, and AA for acrylamide. For the zwitterionic monomer abbreviated SB_x_MA_y_, the spacing between the anionic and cationic functional groups of the zwitterionic unit is x carbon atoms, and the spacing between the zwitterionic unit and the ester or amide bond linking the polymer backbone is y carbon atoms (Table 1).

## 3. Mechanical Reinforcement Methods

In order to improve the mechanical properties of hydrogels, it is necessary to analyze the microscopic behavior of hydrogels in the process of stress application for a breakthrough. The structural collapse of hydrogels begins with the appearance of cracks [34]. Because of the difference in reactivity among the components in the polymerization process, the hydrogels composed of inhomogeneous networks have a stress concentration region composed of short polymer chains [35]. In the loading process, the polymer chains in the stress concentration region become the “weakness” of the hydrogel, which first failed as a crack [36]. Although the “fracture process zone” at the crack tip can dissipate energy through nonlinear elastic response, the potential energy transferred still exceeds the energy needed for the formation of the new interface (fracture energy threshold), resulting in the destruction of the whole structure of the hydrogel [37]. Therefore, the improvement of mechanical properties of hydrogels mainly focuses on two aspects: eliminating the “weakness” in polymer networks and avoiding crack expansion.

### 3.1. Eliminating the Weakness in Polymer Networks

**Figure 1:**
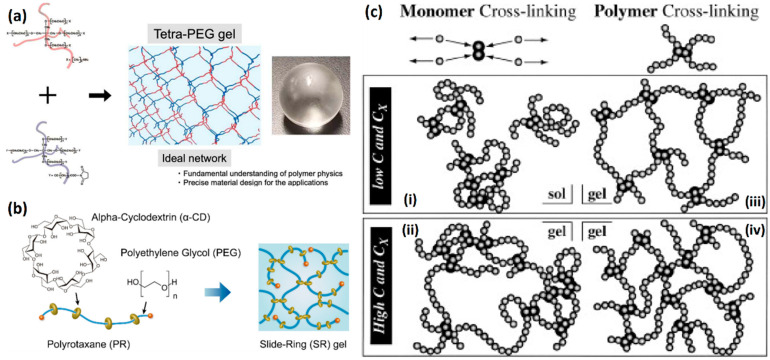
Methods to strengthen hydrogels with the homogenous network: (**a**) tetra- PEG ion gels are prepared with mutually reactive amine and activated ester end groups; reproduced with permission from [41]; (**b**) slide-ring hydrogel prepared with linear polymer axis PEG and ring-shaped alpha-cyclodextrin. Reproduced with permission from [44]. Copyright 2021 Elsevier Ltd. All rights reserved; (**c**) radiation cross-linked hydrogel prepared by γ-ray with better network distribution at both low and high monomer concentration, C, and cross-linking density, C_x_. Reproduced with permission from [48]. Copyright 2002 Elsevier Science Ltd. All rights reserved.

Reducing the randomness of radical polymerization is an effective scheme to minimize the stress concentration region in the hydrogel network. The homogeneity of the hydrogel network can be enhanced by prefabricating the polymer with a definite structure and then assembling it into hydrogel by a covalent bond. Polymers that meet the requirements of uniform molecular weight distribution are prepared first and then assembled by molecular couplings, such as tetra-polyethylene glycol (PEG), to exhibit extremely low network heterogeneity (Figure 1a) [38,39,40]. In the theoretical model, the tetra-PEG hydrogels are only bound by the covalent coupling region. However, in practice, the polymer is still entangled, which leads to the deviation from the ideal model [41]. In addition, the need to prepare homogeneous macromonomers also limits the practical application of tetra-PEG hydrogels. Based on cyclic molecular “pulleys” and shaft chains, the slide-ring gel exhibits excellent tensile strain and volumetric weight variation (Figure 1b) [42,43,44]. Slide-ring gel is mainly used in soft electronic equipment. The conformational change between ring and chain has a decisive effect on the mechanical properties of glide ring gel. Reducing the covering area of the ring and increasing the length of the shaft chain can improve the toughness [45,46]. Because of the existence of the ring-chain, the selection of monomer is also limited, and the influence of conformation change on mechanical properties leads to more complex mechanical behavior. 

In addition, irradiated cross-linked hydrogels have better network homogeneity than chemical cross-linked hydrogels because the high energy of the irradiated sources eliminates the difference in reaction activity (Figure 1c) [47,48]. In addition, higher irradiation doses can also kill bacteria, allowing hydrogels to be applied under conditions that require strict asepsis [49]. However, irradiation can cause degradation of natural polymer hydrogels, resulting in loss of mechanical properties and structural stability. Therefore, the effect of high-energy irradiation on polymer structure remains to be further studied. In addition to irradiation, controllable radical polymerization has also been applied to the preparation of hydrogels to obtain better polymer chain distribution and uniformity. The hydrogels prepared by reversible addition–fragmentation chain transfer polymerization (RAFT) have better mechanical properties than those prepared by ordinary radical polymerization. Because of the existence of a disulfide structure, the hydrogels often have color and uncomfortable smell [50].

Uncontrollable entanglement between polymer chains is not expected in the preparation of homogeneous network hydrogels, which makes hydrogels break rapidly and have no self-healing ability. Compared with the homogeneous network hydrogels designed to eliminate the “weakness” of the network, the hydrogels designed for enhancing the energy dissipation pay more attention to how to avoid the crack tip extension and overall failure of the hydrogels [51].

### 3.2. Avoiding Crack Expansion

**Figure 2:**
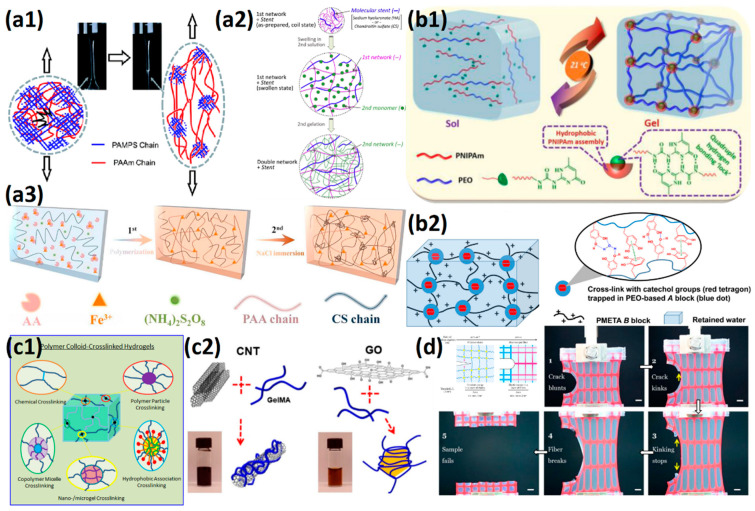
Methods to strengthen hydrogels based on enhancing mechanical energy dissipation: (**a1**–**a3**) double network hydrogels with rigid chemical cross-linked polyelectrolyte 1st network and soft 2nd network (**a1**), hydrophilic molecular stent filled both neutral 1st and 2nd networks (**a2**) and non-covalent cross-linked 1st and 2nd networks (**a3**). Reproduced with permission from [5] (Copyright 2010, Royal Society of Chemistry), [55] (Copyright 2016 Acta Materialia Inc. Published by Elsevier Ltd. All rights reserved) and [58] (Copyright 2018 Elsevier B.V. All rights reserved), respectively; (**b1**,**b2**) dual-cross-linked hydrogel with hydrogen bond (**b1**) and π–π stacking (**b2**). Reproduced with permission from [62] (Copyright 2017, American Chemical Society) and [63] (Copyright 2017, American Chemical Society); (**c1**,**c2**) functional cross-linking sites cross-linked hydrogel with polymer units (**c1**) and nanofillers (**c2**). Reproduced with permission from [66] (Copyright 2018 Wiley Periodicals, Inc.) and [69] (Copyright 2019, American Chemical Society); (**d**) scaffold-reinforced elastomer with macroscopic fibers. Reproduced with permission from [70]. Copyright 2019 Elsevier Ltd. All rights reserved.

Double-network hydrogel(DN) design is a traditional way to enhance the mechanical energy dissipation of hydrogels (Figure 2a1–a3) [52]. The highly swollen rigid first network is broken under excessive stress, and the flexible second network conducts and dissipates mechanical energy. The fractured unit in a rigid network is called a “sacrifice bond” [5]. Double-network hydrogels have developed rapidly over the past two decades due to the combination of both the rigidity of the first network and the toughness of the second network [8,53,54]. The introduction of molecular stents solved the limitation that the first network needs high swelling polyelectrolyte monomers [55,56]. The first network with a dynamic covalent bond as the sacrificial bond also compensates for the permanent mechanical damage in the first network composed of chemical covalent sacrificial bonds, leading to better self-healing properties [57,58]. Dual-cross-link hydrogel not only has the ability to dynamically regulate the distribution of polymer chains but also has the ability to convert mechanical energy to thermal energy by further enhancing the interaction between polymer chains in the network, thus exhibiting high toughness [59,60]. Structures with hydrophobic interaction, π–π stacking, dipole interaction, hydrogen bond interaction, and electrostatic interaction are the most common strengthening elements (Figure 2b1,b2) [61,62,63]. The introduction of strong noncovalent interaction units also gives hydrogels excellent self-healing capabilities [64,65]. In addition, replacing 0-dimensional covalently cross-linked dot and non-covalently cross-linked dot with multi-dimensional structures can deliver more value to hydrogel (Figure 2c1,c2) [66]. Microgel cross-linking sites provide more capacity for elastic potential energy storage, mechanical energy dissipation, and synergistic enhancement of polymer mechanical energy transmission, leading to four times mechanical energy dissipation efficiency [67]. The macromolecular polymer microspheres cross-linking sites provide more cross-linking sites and connect a large number of long and homogeneous polymer chains to control the cross-linking density and distance. The destruction and recombination of physical interactions within microspheres also provide additional mechanical energy dissipation capabilities [68]. Nanostructured fillers can not only store energy, dissipate mechanical energy, and reconstruct polymer networks through the adsorption–desorption process but also endow hydrogels with unique electrical, magnetic, and optical properties [12,69]. As a biomedical engineering material, soft hydrogels and rigid skeleton composites based on biomimetic hydrogels, such as muscles and tendons, exhibit advantages in resisting crack propagation. Scaffold-reinforced hydrogels often exhibit high toughness and crack resistance as cracks propagate through the soft hydrogel to the hard skeleton, which provides strong mechanical energy dissipation capacity, and limits crack propagation (Figure 2d) [70].

## 4. Recent Advances in Mechanical Reinforced Zwitterionic Hydrogels

Due to the brittleness of the anti-polyelectrolyte effect, zwitterionic hydrogels cannot exhibit their super anti-fouling properties under physiological conditions. Therefore, the improvement of mechanical properties of zwitterionic hydrogels is not only the enhancement of mechanical energy dissipation or elimination of “weakness” units but also the inhibition of the anti-polyelectrolyte effect. Meeting the requirements of anti-fouling performance or ion channel requirements is an essential principle during the mechanical strengthening of zwitterionic hydrogels.

### 4.1. Multiple Network Hydrogels

As an electrically neutral polymer, the zwitterionic polymer network is soft and stretchable in deionized water, so it is often used as part of the second network in the design of double network hydrogels [71]. Flexible zwitterionic networks with high molar concentrations provide good anti-fouling properties. In contrast, the first network with a high cross-linking degree provides hydrogel rigidity [72,73]. It is noteworthy that zwitterionic polymers often require the addition of cross-linking agents to form interpenetrating networks with the first network due to the lack of physical interaction forces (such as hydrogen bonds, hydrophobic interactions, etc.) that can form sufficient entanglement with the other network [74].

Yin et al. [75] prepared zwitterionic double-network hydrogels with polyelectrolyte poly(2-acrylamido-2-methylpropanesulfonic) (pAMPS) as the first network and zwitterionic pCB_1_MA_2_ as the second network. The equilibrium water content of pAMPS/pCB_1_MA_2_ double network hydrogel was 78.7%, which was significantly lower than that of pCB_1_MA_2_ hydrogel at the same molar concentration. DN hydrogels showed the tensile strain of 6.0 mm/mm, tensile fracture stress of 1.4 MPa, tensile modulus of 0.9 MPa, and work of extension of 2.4 MJ/m^3^ much higher than those of single network pCB_1_MA_2_ hydrogel. The excellent resistance to macrophage adhesion was also maintained. Huang et al. [76] prepared DN hydrogels with cationic monomer (trimethylamino)ethyl methacrylate chloride (TMAEMA) as the first network and zwitterionic sulfobetaine vinylimidazole (SBVI) as the second network. The final hydrogel pTMAEMA/pSBVI exhibited a stable swelling ratio that did not vary with ion concentration. Zwitterionic pTMAEMA/pSBVI3 hydrogels prepared with 1 M TMAEMA and 3 M SBVI showed tensile stress of 0.53 MPa, tensile strain of 2.0 mm/mm, and Young’s modulus of 105 KPa in water, which were 120, 5.7, and 10.5 times of the single network pTMAEMA hydrogels, respectively. Although the swelling ratio of zwitterionic double network hydrogels did not change significantly with ionic strength in solution, there is still a tensile strain decrease (2.0 mm/mm in 0 M to 0.7 mm/mm in 1 M NaCl) and an increase in Young’s modulus (105 KPa in 0 M to 300 KPa in 1 M NaCl). It is worth noting that zwitterionic hydrogels exhibited tensile stress of 0.85 MPa and tensile strain of 1.8 mm/mm in PBS solution, which has a good application prospect in the biomedical field. Zou et al. [77] prepared triple network hydrogels with glutaraldehyde cross-linked chitosan as the first antimicrobial network, zwitterionic pSB_3_MA_2_ polymer as the second anti-fouling network, and poly(2-hydroxyethyl acrylate) (pHEA) as the third mechanical to strengthen the network. The triple network hydrogel exhibited excellent toughness at 75% equilibrium water content, with compressive stress of 82 MPa at 95% compressive strain and 0.4 MPa at 10.0 mm/mm tensile strain. In addition to the classical first rigid network and the second flexible zwitterionic network, the double network hydrogels with interpenetrating non-covalent cross-linking network and zwitterionic polymer network have also been widely studied. Interpenetrated network (IPN) hydrogels composed of cellulose nanofiber networks and zwitterionic pSB_3_MA_2_ networks exhibited 0.12 MPa tensile stress, 9.2 mm/mm tensile strain, and 24.5 KPa Young’s modulus [29]; hydrogels composed of alginate-Ca^2+^ networks and zwitterionic pCB_1_AA_3_ networks exhibited tensile stress 0.69 MPa, tensile strain 3.3 mm/mm, elastic modulus 0.28 MPa, and also excellent self-healing properties [78]. 

**Figure 3:**
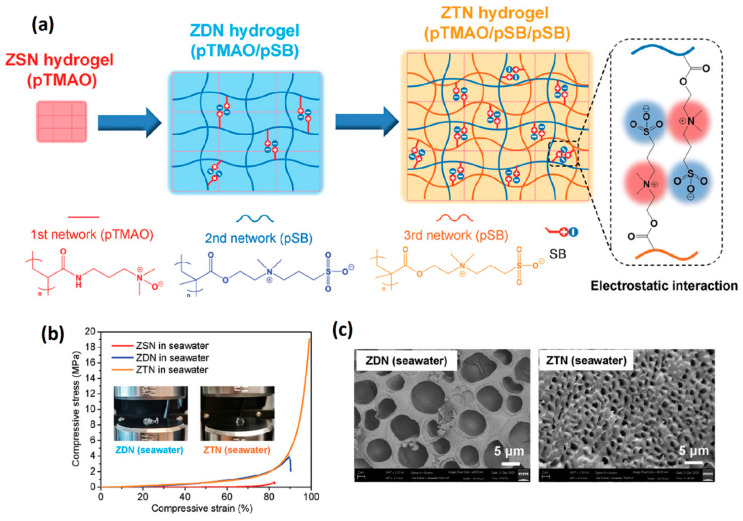
Mechanical strengthening pure zwitterionic hydrogels prepared by multiple networks: (**a**) schematic illustration of ZTN hydrogel with 1st pTMAO network and double pSBMA networks; (**b**) uniaxial compression test of pure zwitterionic hydrogels swelling in seawater. The introduction of the 3rd network further increased both compressive stress and toughness; (**c**) SEM images of multiple network pure zwitterionic hydrogels swelling in seawater. The introduction of the 3rd network further enhances dipole–dipole interaction between zwitterionic units leading to a more compact structure. Reproduced with permission from [81]. Copyright 2021 Wiley-VCH GmbH.

The purely zwitterionic double network hydrogel assembled by multiple zwitterionic polymer networks has more potential in the application where the performance of anti-fouling is strictly required or needs to be kept for a long time. Huang et al. [79] interpenetrated the second network, pSB_3_AA_3_, into the first zwitterionic network, poly(lysine acrylamide) (pLysAA), under acid conditions (pH = 2). The protonated carboxyl groups of the LysAA units provide additional hydrogen bond interactions that enhance the mechanical properties of the double network hydrogels even after swelling in PBS solution at pH = 7 (1.05 MPa tensile stress and 1.1 mm/mm tensile strain). Hydrogels exhibited excellent anti-fouling properties due to a fully zwitterionic network. Subcutaneous 30 days implantation showed no capsule formation between the hydrogels and tissue. Pure zwitterionic elastic network (ZEN) hydrogels with 62% equilibrium water content were prepared using CB_2_AA_3_ as a highly swollen elastic network and SB_3_MA_2_ as a loose viscous network [80]. ZEN hydrogels achieved compressive stress of 22.3 MPa at 99% compressive strain and kept the original shape after stress release. During the tensile process, ZEN hydrogel also exhibited 1.7 mm/mm tensile strain and 1 MPa tensile stress. In addition, ZEN hydrogels could effectively resist the formation of fibrous capsules after implantation in animals for one year, further confirming the value of zwitterionic hydrogels under physiological conditions. Li et al. [81] used poly(trimethylamine N-oxide) (pTMAO) with stronger hydrophilicity as the first network, followed by the introduction of double self-associated pSB_3_MA_2_ networks to prepare pure zwitterionic triple network hydrogels (ZTN). Compared with the double network hydrogel, the swelling of ZTN hydrogel in a seawater environment was further limited, the equilibrium water content was 73%, and the ZTN hydrogel could endure the compressive stress of 19 MPa at 99% compressive strain, which was 31 times the single network pTMAO hydrogel and five times the pTMAO/pSB double network hydrogel, respectively (Figure 3). The design of multiple network hydrogels not only ensured the anti-fouling performance of pure zwitterionic hydrogel, but also enhanced the mechanical properties in an ionic solution, which might provide a feasible solution for the practical application of zwitterionic hydrogels.

### 4.2. Dual-Cross-Linked Hydrogels

Dual-cross-linked zwitterionic hydrogels exhibit more physical interactions, including hydrophobic interactions [82,83,84], hydrogen bonds [85,86,87], and π–π stacking [88,89,90]. In an ionic solution, the newly added physical interaction can compensate for the loss of mechanical properties caused by the damage of dipole interaction due to further hydration. The methods for preparing the dual-cross-linked hydrogels include: (1) introducing new physical interaction groups by the molecular design of zwitterionic units and (2) introducing comonomers with physical interaction ability.

#### 4.2.1. Zwitterionic Hydrogels Prepared by Molecular Design

For zwitterionic hydrogels that need to maintain long-term anti-fouling requirements in complex physiological environments, the non-specific adsorption behavior of proteins induced by comonomers is considered [80,91]. The molecular design of zwitterionic units and the introduction of functional groups with effective physical interactions have become effective schemes for strengthening pure dual-cross-linked zwitterionic hydrogels. Introducing functional groups with hydrogen bonding interaction or π–π stacking is the most common method (Table 2). With molecular dynamic simulation, introducing hydroxyl groups between the cationic and anionic groups of the zwitterionic unit can reduce the equilibrium water content and improve compressive strength [92]. When one methyl group of cationic quaternary ammonium salt is replaced by a hydroxyl group, the compressive strength and strain of zwitterionic hydrogel increase obviously [93]. However, when both methyl groups were replaced by the hydroxyl group, the compressive strength of zwitterionic CB_1_MA_2_-OH_2_ hydrogels was almost the same as that of CB_1_MA_2_ hydrogels, with only a tiny increase in compressive strain [93]. This may be due to the effect of steric hindrance, which results in the shielding of dipole–dipole interaction between zwitterionic units. In addition, the increase of the steric hindrance around the quaternary ammonium results in a decrease in the anti-fouling performance of the zwitterionic hydrogel. Therefore, in the molecular design of zwitterionic unit molecules to enhance the work of hydrogels, the maintenance of the dipole effect is the basic principle. 

**Figure 4:**
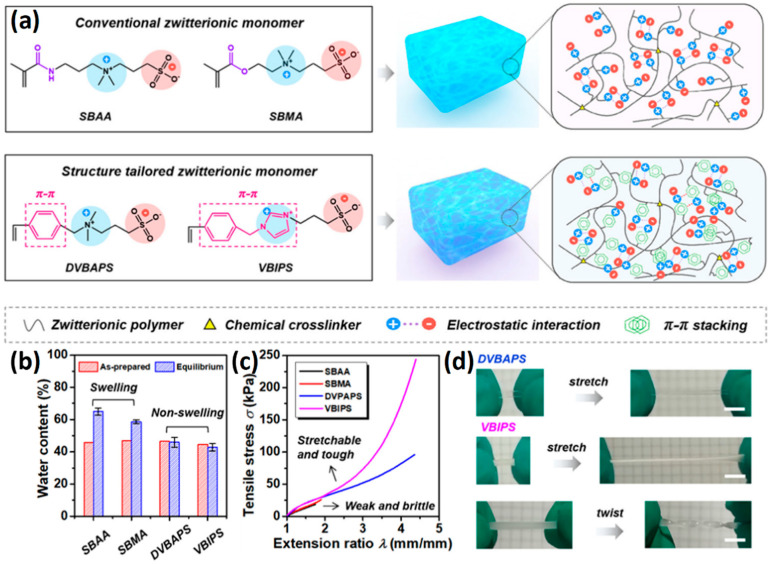
Mechanical strengthening of pure zwitterionic hydrogel based on the molecular design of the zwitterionic unit: (**a**) schematic illustration for the preparation of zwitterionic hydrogels with π–π stacking; (**b**) water content of pure zwitteironic hydrogels. The introduction of π–π stacking significantly restricts further swelling of hydrogel from the as-prepared state to the equilibrium state; (**c**) tensile stress–strain curves of pure zwitterionic hydrogels. Dual-cross-linked pure zwitterionic hydrogels with π–π stacking was both stretchable and tough; (**d**) stretchability of dual-cross-linked DVBAPS and VBIPS hydrogels. Reproduced with permission from [95]. Copyright 2021, American Chemical Society.

The introduction of functional groups with the ability of π–π conjugation between the zwitterionic unit and the polymer backbone can limit the swelling behavior of the zwitterionic hydrogel and improve its toughness. Based on carboxybetaine CB_1_AA_3_ and sulfobetaine SB_3_AA_3_, Liu et al. [94] added triazole groups between amide and zwitterionic units. Unlike the brittleness of CB_2_MA_2_ hydrogel in PBS solution (tensile strain 0.1 mm/mm, compressive strain 50%, Young’s modulus 153 KPa), triazole carboxybetaine acrylamide (TR-CB) and triazole sulfobetaine acrylamide (TR-SB) hydrogels exhibit better toughness at the same monomer concentration and cross-linking density, in which compressive strain increased to 79% and 89%, respectively, tensile strain increased to 0.9 mm/mm and 2.5 mm/mm, and Young’s modulus decreased to 20 KPa and 15 KPa. In addition, TR-CB and TR-SB exhibited compressive fracture stress over 1 MPa and tensile fracture stress over 10 KPa, which were 2.5 and 1.3 times of CB_2_MA_2_, respectively. The stronger dipole–dipole interaction between zwitterionic SB_3_ units makes TR-SB exhibit the best toughness. Tough rat islet encapsulated TR-SB showed 30 days blood glucose concentration control after transplantation due to the mitigating foreign body response. Zheng et al. [95] prepared zwitterionic dimethyl-(4-vinylphenyl)ammonium propane sulfonate (DVBAPS) hydrogels by introducing an aromatic structure between the zwitterionic SB_3_ unit and the polymer backbone. Zwitterionic DVBAPS hydrogels retain the water content of the as-prepared state without further swelling (non-swelling) and exhibit 3.7 mm/mm extension, 80 KPa tensile stress, Young’s modulus of 53 KPa, and over 20 times higher toughness than that of SB_3_AA_3_ hydrogel. The zwitterionic units composed of the conjugated N-atom heterocyclic ring and the anionic functional group exhibit both dipole–dipole interaction and π–π stacking [76,96]. The zwitterionic QTR-CB hydrogel with quaternarized triazole group as a cationic functional group exhibited tensile strain of 0.7mm/mm and compressive strain of 78% in PBS solution, which showed tougher than CB_2_MA_2_ in ionic solution [94]. The 3-(1-(4-vinylbenzyl)-1H-imidazol-3-ium-3-yl)propane-1sulfonate (VBIPS) hydrogels with both N-heterocyclic cations and aromatic structures exhibited a stronger π-π stacking effect, exhibiting a non-swelling effect similar to DVABPS and a toughness 40 times greater than that of SB_3_AA_3_ hydrogels, which overcame the trade-off between electrolyte swelling and mechanical property. (Figure 4) [95].

**Table 2:** Molecular design of zwitterionic hydrogels and their mechanical properties.

Dual-Cross-Linking	Zwitterionic Units	Components	Mechanical Properties	Ref.
Hydrogen bond	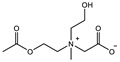	CB_1_MA_2_OH-1: 3M Crosslinker: 2 mol% EWC: 71.7%	Compressive strain: 55% Compressive stress: 0.75 MPa	[93]
	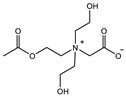	CB_1_MA_2_OH-2: 3M Crosslinker: 2 mol% EWC: 73.7%	Compressive strain: 50% Compressive stress: 0.45 MPa	[93]
	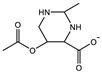	Ectoine: 4.4 M Crosslinker: 1.5 wt% EWC(PBS): 82.0%	Compressive stress: 0.32 MPa Compressive strain: 62% Compressive modulus: 0.17 MPa	[97]
	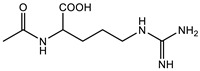	Marg: 0.4 M Crosslinker: 1 wt% EWC: 88.9%	Compressive modulus: 0.21 MPa	[98]
π–π stacking	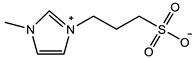	VIPS: 3M Crosslinker: 0.02 mol% EWC: 50.0%	Elastic modulus: 18 KPa Tensile strain: 6.0 mm/mm Tensile stress: 90 KPa	[76]
	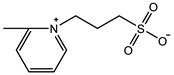	SPV: 2.4 M Crosslinker: 3.0 wt% EWC: 61.0%	Elastic modulus: 105 KPa Elastic modulus (KSCN): 36.5 KPa	[96]
	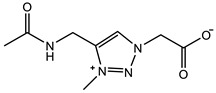	QTR-CB: 2 M Crosslinker: 5.0 mol% Swelling in PBS	Compressive strain: 78% Compressive stress: 0.78 MPa Tensile strain: 0.7 mm/mm Tensile stress: 0.013 MPa Young’s modulus: 30 KPa	[94]
	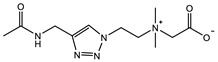	TR-CB: 2 M Crosslinker: 5.0 mol% Swelling in PBS	Compressive strain: 79% Compressive stress: 1.0 MPa Tensile strain: 0.9 mm/mm Tensile stress: 11 KPa Young’s modulus: 20 KPa	[94]
	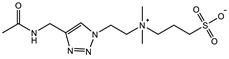	TR-SB: 2 M Crosslinker: 5.0 mol% Swelling in PBS	Compressive strain: 89% Compressive stress: 1.05 MPa Tensile strain: 2.5 mm/mm Tensile stress: 13 KPa Young’s modulus: 15 KPa	[94]
	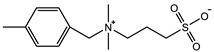	DVBAPS: 4 M Crosslinker: 0.5 mol% EWC: 48.4%	Tensile strain: 3.7 mm/mm Tensile stress: 75 KPa Young’s modulus: 53 KPa	[95]
	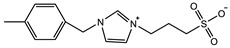	VBIPS: 4 M Crosslinker: 0.5 mol% EWC: 44.2%	Tensile strain: 4.4 mm/mm Tensile stress: 200 KPa Young’s modulus: 56 KPa	[95]

#### 4.2.2. Zwitterionic Ionic Hydrogels Prepared by Multicomponent Copolymerization

Although the mechanical and anti-fouling properties of zwitterionic hydrogels can both be considered by the molecular design of zwitterionic units, this also leads to the complexity of monomer synthesis and the functional limitation of hydrogels. The preparation of zwitterionic hydrogels by copolymerization has a simple process and great potential for functionalization. Although the anti-fouling performance would be compromised, it still has significant research value in the application where it is not necessary to keep strict anti-fouling performance for a long time. 

**Figure 5:**
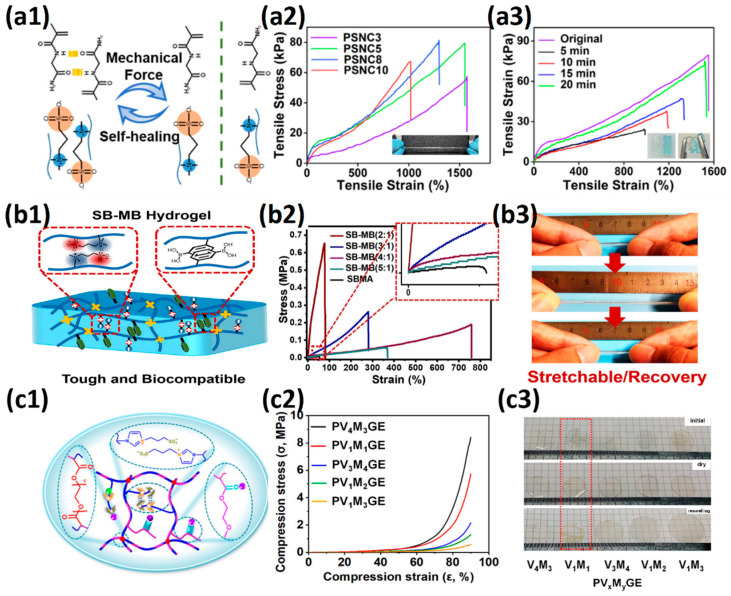
Mechanical strengthening of zwitterionic hydrogels prepared by copolymerization: (**a1**–**a3**) zwitterionic hydrogel was prepared with NAGA as a copolymerization component. The dipole–dipole interaction between zwitterionic units and the hydrogen bond interaction between NAGA units synergistically promote the mechanical properties and self-healing ability of hydrogels (**a1**), leading to stretchable, tough (**a2**) and rapid self-healing (**a3**). Reproduced with permission from [99]. Copyright 2021, American Chemical Society; (**b1**–**b3**) zwitterionic hydrogel prepared with MPBA as a copolymerization component. The aromatic ring of phenylboronic acid provided additional π–π stacking (**b1**). The introduction of the MPBA component improved both the toughness and modulus of zwitterionic hydrogels (**b2**) and also enabled the hydrogels to exhibit good recovery ability (**b3**). Reproduced with permission from [102]. Copyright 2021 Elsevier Inc. All rights reserved; (**c1**–**c3**) zwitterionic hydrogel with oleophilic elastomer pMEA segments (**c1**). When the weight ratio of zwitterionic VIPS/oleophilic MEA was 1/1, the prepared PV_1_M_1_GE showed 6 MPa at 90% compressive strain (**c2**) and non-swelling in electrolytes (**c3**). Reproduced with permission from [33]. Copyright 2021, American Chemical Society.

The copolymer with a strong hydrogen bond can provide hydrogen bond interaction and hydrogen bond–dipole interaction for hydrogels, and the hydrated layer on a copolymer can also provide nonspecific protein adsorption resistance, leading to a low-fouling hydrogel. Huangfu et al. [99] prepared the copolymer hydrogel with 16 mm/mm tensile strain, 77.5 KPa tensile stress and 1.9 KPa tensile modulus using a strong hydrogen bond monomer N-(2-amino-2-oxyethyl)acrylamide (NAGA) and zwitterionic SB_3_MA_2_ as a wound dressing (Figure 5a1–a3). The introduction of NAGA enables the hydrogel to produce sufficient adhesion with the skin wound and accelerates the wound healing. The p (HEAA-co-SB_3_AA_3_) hydrogel prepared by Zhang et al. [100] exhibited 34 mm/mm tensile strain and 0.17 MPa tensile stress. After introducing the conductive polymer PEDOT: PSS, which interacted with zwitterionic polymers by electrostatic interaction, as the interpenetrating network, the hydrogel exhibited 50 mm/mm tensile strain and 0.34 MPa tensile stress. The integration of zwitterionic pSBAA and conductive PEDOT: PSS promoted charge transfer through the optimized conductive path and further acted as a fully polymeric biosensor. Yang et al. [101] also introduced 2-Hydroxyethyl methacrylate (HEMA) with hydrogen bonding ability and 2-(dimethylamino) ethylacrylatemethochloride (DAC) with electrostatic bonding ability to copolymerize with zwitterion SB_3_MA_2_. When the molar ratio of the three components was HEMA:DAC:SBMA = 2:4:4, the copolymer hydrogel exhibited tensile strain of 6.8 mm/mm, tensile stress of 330 KPa, and optimum self-healing efficiency (96.5% at room temperature for 10 h). 

It is also a general method to inhibit the swelling ability of zwitterionic hydrogels and enhance the mechanical properties by using the comonomers with π–π conjugation and hydrophobic interaction. Guo et al. [102] prepared a tough hydrogel by copolymerizing zwitterionic SB_3_MA_2_ with glucose-responsive (3-methacrylamidophenyl) boronic acid (MPBA) in a water/DMSO mixed solution. When SB:MB = 4:1, the hydrogel exhibited 7.5 mm/mm tensile strain, 180 KPa tensile stress and 0.6 KPa elastic modulus, and the hydrogel was able to withstand 7 MPa compressive stress at 78% compressive strain (Figure 5b1–b3). The prepared zwitterionic aromatic skin showed both highly sensitive pressure and glucose detection. Liu et al. [33] polymerized into heterogeneous copolymer networks in lithium electrolytes using zwitterionic monomer 3-(1-vinyl-3-imidazolio)-propanesulfonate (VIPS) as hydrophilic component and 2-methoxyethyl acrylate(MEA) as a lipophilic component. The prepared gel electrolyte PVMGE exhibited non-swelling properties after soaking in the electrolyte for a long time and exhibited compressive stress of 5.8 MPa at 90% compressive strain and high ion conductivity of 1.78 mS/cm (Figure 5c1–c3). 

In addition to non-covalent interactions, excellent self-healing properties can also be achieved by introducing dynamic covalent bonds in copolymerized zwitterionic hydrogels. He et al. [103] prepared copolymerized zwitterionic hydrogel with VIPS and acrylate acid (AA), which could form active coordination covalent bonds with multivalent ions. The as-prepared hydrogel could withstand 49% compressive strain and 21.6 MPa compressive stress in Fe^3+^ ion solution and 70% compressive strain and 12.6 MPa compressive stress in Ca^2+^ ion solution. Chen et al. [104] prepared two kinds of zwitterionic copolymers by copolymerizing zwitterionic 2-methacryloyloxyethyl phosphorylcholine (MPC) with 5-methacrylamido-1,2-benzoxaborole (MAABO) and dopamine methacrylamide (DMA), respectively. Then, the hydrogels assembled by two zwitterionic copolymers showed excellent self-healing properties by using the dynamic covalent bonds of borate esters.

### 4.3. Functional Cross-Linkers Reinforced Zwitterionic Hydrogels

In addition to designing interactions in polymer chains, the elevated dimensions of cross-linking sites that have long been regarded as “dots” are also beneficial to the mechanical properties of hydrogels [105]. In the mechanical enhancement of zwitterionic hydrogels, functional cross-linking sites can also be divided into 0 to 3 dimensions. The 0D cross-linking site is considered to be a rigid filler with additional energy storage capacity and the ability to promote the redistribution of flexible networks and mechanical dissipation. Polystyrene resin H103 was used as filler to prepare zwitterionic hydrogels CB_2_MA_2_ and SB_3_MA_2_, respectively [106]. The compressive modulus of hydrogel (0.044 MPa to 0.281 MPa for SB_3_MA_2_ hydrogel and 0.070 MPa to 0.265 MPa for CB_2_MA_2_ hydrogel) and compressive fracture strength (1.76 times for SB_3_MA_2_ hydrogel and 2.19 times for CB_2_MA_2_ hydrogel) were significantly increased by adding H103 rigid resin. Li et al. [22] also used MOF-based MIL101 (Cr) to prepare CB_2_MA_2_ hydrogels, increasing their compression modulus from 0.062 MPa to 1.070 MPa and compressive fracture stress from 0.10 MPa to 1.38 MPa. More importantly, based on the strong anti-fouling ability of zwitterionic hydrogels, the introduced 0D nanoparticles mentioned above can act as effective protein-bound toxin adsorbents without protein blocking adsorption sites even in 100% fetal bovine serum.

**Figure 6:**
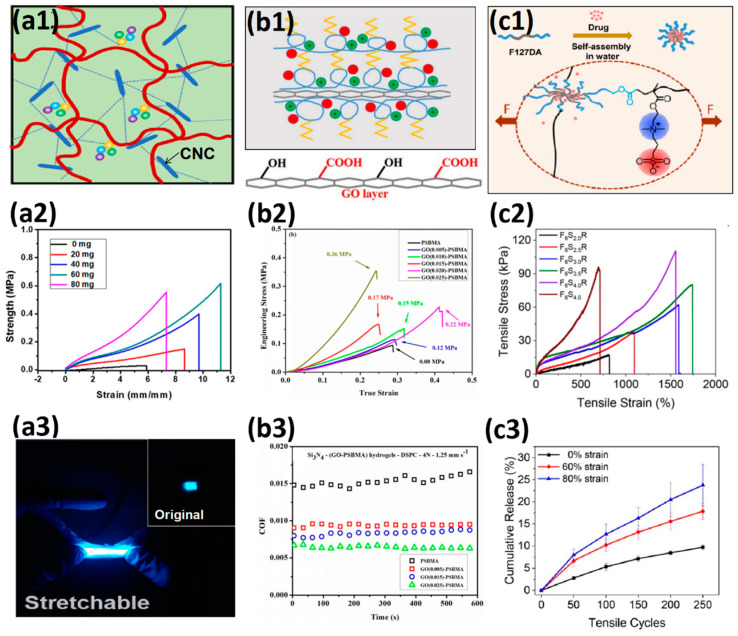
Mechanical strengthening of zwitterionic hydrogels prepared with the functional cross-linker: (**a1**–**a3**) zwitterionic hydrogel with 1D CNC nanotubes (**a1**) showed enhanced tensile strain and toughness of zwitterionic hydrogels (**a2**) and further acted as an electroluminescent display (**a3**). Reproduced with permission from [107]. Copyright 2019, American Chemical Society; (**b1**–**b3**) zwitterionic hydrogels with 2D graphene oxide nanosheets (**b1**) showed almost 5 times more stress than the original pSBMA hydrogel (**b2**). The introduction of graphene oxide nanosheets further decreased COF from 0.015 to 0.006 between Si_3_N_4_ ball and hydrogel using DSPC liposomes as lubricants (**b3**). Reproduced with permission from [110]. Copyright 2019, American Chemical Society; (**c1**–**c3**) zwitterionic hydrogels with 3D Pluronic F127DA micelles (**c1**) showed more than 1500% tensile strain and over 100 KPa tensile stress (**c2**). Mechano-responsive drug release was observed at strain-limited tensile cycles (**c3**). Reproduced with permission from [111]. Copyright 2020, American Chemical Society.

The 1D cross-linking sites are rod-shaped or tube-shaped. These fillers provide highly ordered structures that enhance the tensile properties of hydrogels and resist fracture extension. Yang et al. [107] introduced 60 mg/mL cellulose nanocrystals (CNC) into the 3D printing ink to prepare SB_3_MA_2_-co-AAm hydrogel(Figure 6a1–a3). Hydrogels showed a tensile stress of 0.61 MPa, a tensile strain of 11.3 mm/mm, and the Young’s modulus of 54 KPa, which were 20 times, 2 times, and 7.7 times the original hydrogels without CNC, respectively. Lai et al. [108] introduced cationic cellulose nanocrystals (CCNC) and Al^3+^ into copolymer hydrogels of SB_3_MA_2_ and AA. The stiffness of hydrogels was further enhanced by the addition of COO^−^-Al^3+^ dynamic coordination covalent bond and COO^−^-CCNC^+^ electrostatic interaction. Hydrogels with 7 wt% CCNC showed tensile strain 11.9 mm/mm, tensile stress 0.66 MPa, Young’s modulus 0.14 MPa, and toughness 4500 KJ/m^3^, which were 0.85 times, 1.94 times, 1.4 times and 1.5 times the 7 wt% of CNC filled hydrogels, and 0.50 times, 3.67 times, 3.5 times, and 1.8 times of non-nano-filled hydrogels, respectively. Due to the formation of a stronger physical network in water, the nanofiller concentration needed for reinforcing and 3D printing was reduced, leading to better transparency.

The 2D cross-linking sites are nanosheets with large specific surface areas, which exhibit higher mechanical energy storage and dissipation capacity. Pei et al. [109] prepared tissue adhesive polydopamine (PDA) loaded laponite composited zwitterionic SB_3_MA_2_ hydrogel, exhibiting tensile strain of 8.0 mm/mm and tensile stress of 0.09 MPa and being able to withstand compressive stress of 6 MPa at 90% compressive strain. The introduction of PDA-loaded laponite endowed zwitterionic hydrogel strong adhesion on organs such as the heart, liver and lungs, which leads to high strain sensitivity for monitoring and diagnostics. Wang et al. [110] introduced graphene oxide (GO) into SB_3_MA_2_ hydrogel, significantly increasing the compressive strength (Figure 6b1–b3). The compressive stress of GO reinforced zwitterionic hydrogel increased to 0.36 MPa, which was almost five times the original SB_3_MA_2_ hydrogel. Moreover, due to the synergetic interaction between the zwitterionic network and GO nanosheets, the friction coefficients (COF) decreased to 0.006 and showed better lubrication properties. 

The 3D cross-linking site has a more fully utilized spatial structure, which can not only provide more elastic potential energy storage, but also be used as a mechanical energy dissipation unit. Fang et al. [111] used Pluronic F127DA, a self-assembled micelle of hydrophobic cores in water, as a 3D cross-linking site in the zwitterionic SB_3_MA_2_ network. The hydrogel exhibited 14.2 mm/mm tensile strain, 112 KPa tensile strength, and 1.41 MPa compressive stress at 90% compressive strain. The 3D Pluronic F127 micelle can also release the contained drug as a mechano-responsive delivery system (Figure 6c1–c3). Sun et al. [112] introduced the Pluronic F127DA 3D cross-linking site into the p(HEMA-co-SBMA) copolymer hydrogel. When HEMA:SBMA = 2:3, the hydrogel exhibited a tensile strain of 10.0 mm/mm, a tensile stress of 60 KPa, and a compressive stress of 37 MPa at 98% compressive strain. Li et al. [113] added waterborne polyurethane (PU) emulsion microspheres to the p(SB_3_MA_2_-co-AAm) hydrogel. The PU chains were stretched during stress application, providing additional mechanical energy dissipation and more entanglement and friction with the copolymer network.

### 4.4. Other Reinforcement Method to Prepare Tough Zwitterionic Hydrogel

In addition to the above commonly used methods to enhance the mechanical properties of zwitterionic hydrogels, researchers have also designed more diversified reinforcement strategies. Liu et al. [114] introduced an electrospun skeleton with high toughness to prepare scaffold-reinforced hydrogels based on the high softness and dipole–dipole interaction of zwitterionic polymers. The tensile properties of SRgels were determined mainly by the electrospun skeleton. The ultra-flexibility of zwitterionic hydrogels not only avoids the damage caused by shear force, but also achieves dipole–dipole enhancement due to the pressure by electrospun fibers. The Young’s modulus of SRgel was shown as 2 MPa, similar to the Young’s modulus of artery indicating SRgel might be a potential artificial blood vessel candidate with reliable resistance to coagulation. Fang et al. [115] used the hydrogen bond–dipole interaction between tannic acid and zwitterionic units to enhance zwitterionic SB_3_MA_2_ hydrogel. The toughness of zwitterionic hydrogels increased significantly with the increase of tannic acid concentration. The prepared TA-reinforced zwitterionic hydrogel showed compressive strength at 18.4 MPa and resistance to the compressive cyclic test at 0.2 MPa for up to 3500 cycles. Combined with the antioxidant and antibacterial properties of TA, the prepared zwitterionic hydrogel could be a great candidate for diabetic foot ulcers. Jiang et al. [116] prepared microtubule self-assembled hydrogels with β-CD and TDPS, and showed more than 100 KPa elastic modulus. Each microtubule was composed of 2 TDPS and 3 CD molecules. Moreover, in order to avoid the strong hydration ability of zwitterionic ions in water, organogel [72,117,118] prepared by glycerine–water mixture solution and ionogel [119,120,121] prepared by ionic liquid have also received extensive attention. Zhao et al. [119] prepared p(SBMA-co-AAm) hydrogels in ionic liquid 1-ethyl-3-methylimidazolium dicyanamide ( [EMIM] [DCA]). Ionogels showed tensile stress 200 KPa and tensile strain almost 5.0 mm/mm with 2 mol% PEGDA 1000 cross-linker. As a strain sensor, the prepared ionogel exhibited outstanding sensitivity, low detection threshold, and high durability (1000 cycles at 1.0 mm/mm tensile strain).

## 5. Conclusions and Perspective

Zwitterionic polymers have been widely used in many fields. Zwitterionic hydrogels have great potential in biomedical applications because they are the only materials that can resist fibrous capsule formation after 1 year of implantation. In this work, we reviewed the works for overcoming the mechanical defects of zwitterionic hydrogels in order to realize their practical application. However, the tensile stress of even the toughest zwitterionic hydrogels is still much less than the compressive stress. This is probably due to the lack of forces that can overcome the hydration layer between the zwitterionic units to achieve dipole–dipole interaction during the tensile process. Although the addition of copolymer can improve the tensile properties of zwitterionic hydrogels to some extent, it can also cause the defect of intrinsic zwitterionic anti-fouling properties. As a material with the aim of application, the scheme of mechanical properties enhancement of zwitterionic hydrogels should rely more on the guidance of application scenarios, methods, and frequency. The complexity of the physiological environment also requires more research to ensure the structural stability and long-term effects on animals of various zwitterionic products, including pure zwitterionic hydrogels, multicomponent hydrogels, or hydrogels coating materials. In addition, clinical studies based on zwitterionic hydrogels are still needed. We also need to establish a complete evaluation system to evaluate the balance between anti-fouling and mechanical properties of zwitterionic hydrogels and their ultimate application value. We believe that more practical application-based zwitterionic hydrogels will eventually be introduced and used in various fields with the development of more applications of zwitterionic hydrogels.

## Figures and Tables

**Table 1:** Zwitterionic polymers are commonly used as mechanical energy dissipation units through dipole interaction.

	-R	-R_0_	Abbreviation
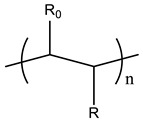	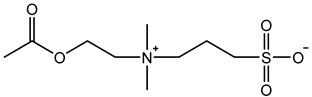	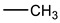	SB_3_MA_2_
	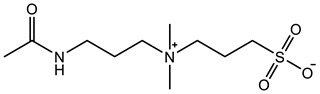	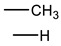	SB_3_MAA_3_ SB_3_AA_3_
	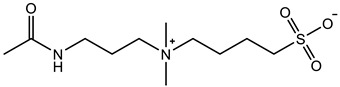	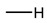	SB_4_AA_3_
	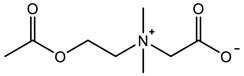	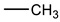	CB_1_MA_2_
	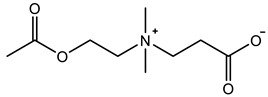	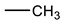	CB_2_MA_2_
	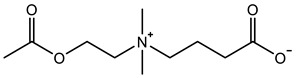	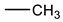	CB_3_MA_2_
	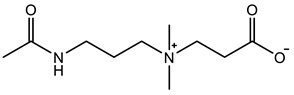	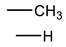	CB_2_MAA_3_ CB_2_AA_3_
	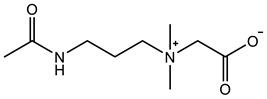	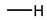	CB_1_AA_3_
	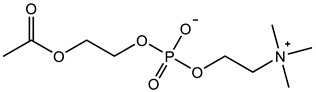	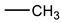	MPC
	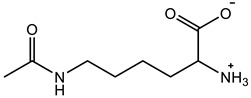	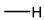	LysAA

## Data Availability

Not applicable.
